# Comparison of ivabradine plus β-blockers versus β-blocker therapy only

**Published:** 2010-04

**Authors:** J Aalbers

**Affiliations:** Special Assignments Editor

## Introduction

A study of patients with stable angina and moderate left ventricular systolic dysfunction has shown that the addition of ivabradine to bisoprolol produced additional anti-anginal and anti-ischaemic effects that were not achieved with up-titration of bisoprolol.^1^ This indicative study is of importance to clinicians as they are frequently faced with patients on β-blockers who are not able to tolerate the full target dose, as defined from evidencebased clinical trials.

Ivabradine is a novel agent that reduces heart rate (HR) by selective and specific inhibition of the If current in sino-atrial cells, leading to prolongation of the slow diastolic depolarisation phase of the action potential.

**Fig. 1.**
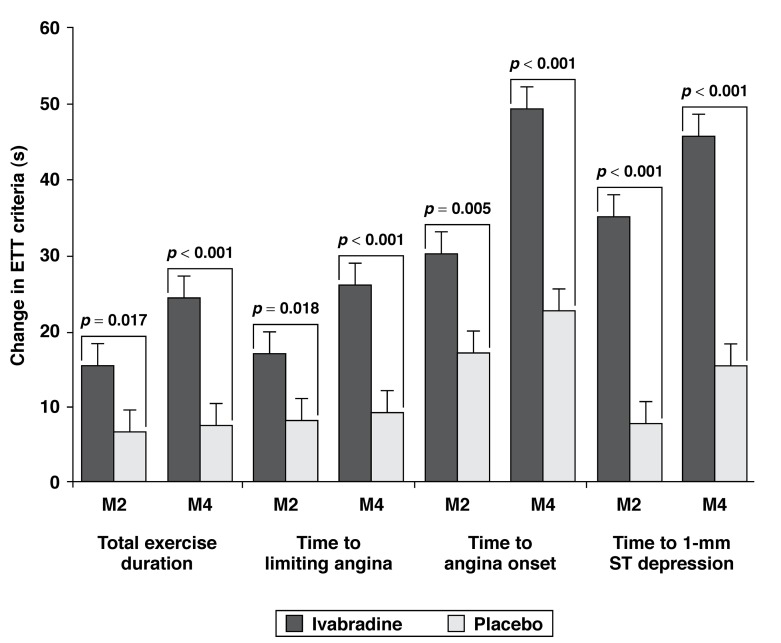
Change in exercise tolerance test criteria between baseline and M2 visit and between baseline and end of study (M4) in the full analysis set.

Placebo-controlled studies in angina patients have shown that ivabradine improves exercise tolerance, lengthens time to ischaemia, and has anti-anginal and anti-ischaemic efficacy similar to that of atenolol or amlodipine. The ASSOCIATE study in stable angina patients receiving the beta-blocker atenolol has demonstrated that ivabradine reduces HR and improves exercise capacity (Fig. 1).

This study, presented at the ACC congress, included 29 patients with chronic stable angina (class II) who had had a myocardial infarction more than three months before and had moderate left ventricular systolic dysfunction on stable therapy, including bisoprolol 5 mg once daily. Therapy included aspirin, and statins enalapril and furosemide in cases with congestive heart failure.

Over a period of two months, resting heart rate was reduced in both groups to similar levels (60 bpm). However more patients in the ivabradine-treated group (7.5 mg twice daily) showed improvements in angina and more patients moved from angina class II to angina class I than those who received the up-titrated bisoprolol dose (10 mg once daily). Importantly, walking distance and exercise tolerance improved in the ivabradine group while no improvement occurred in the bisoprolol-treated group.
